# Application of Lightweight Structure in Automobile Bumper Beam: A Review

**DOI:** 10.3390/ma16030967

**Published:** 2023-01-20

**Authors:** Bing Du, Qichang Li, Changqi Zheng, Suozhu Wang, Cong Gao, Liliang Chen

**Affiliations:** 1 Chongqing Key Laboratory of Nano–Micro Composite Materials and Devices, School of Metallurgy and Materials Engineering, Chongqing University of Science and Technology, Chongqing 401331, China; 2 College of Aerospace Engineering, Chongqing University, Chongqing 400030, China; 3 Innovation Center, Chongqing Polycomp International Corp., Chongqing 401321, China; 4 Chongqing ChangAn Global R&D Center, ChangAn Automobile Co., Ltd., Chongqing 400023, China

**Keywords:** bumper beam, composite material, fabrication, performance

## Abstract

The bumper beam is an important device to ensure the safety of the car, which can effectively alleviate the force and absorb energy when the car collides. Traditional bumper beams are mostly made of high-strength steel, which has high strength and a low production cost but a heavy weight. With the requirement of being lightweight, high-strength steel is not able to meet the needs of lightweight cars, and composite materials have become the answer to the problem of a light weight in cars due to their excellent performance of being lightweight and high strength. This article introduces the case study on materials of bumper beams and presents the application of traditional materials and composite materials in bumper beams. Then, the fabrications and processes of bumper beams, a performance assessment, experimental tests, and a finite element analysis of the bumper beam are carried out. This paper also represents the study of optimization in automobile bumper beams.

## 1. Introduction

The bumper beam is the foremost to be impacted in car collisions, wherefore its parameters are one of the most important norms for evaluating the safety and reliability of vehicles. In the event of a collision, the impact force first acts on the bumper beam composed of the energy-absorbing box, the main beam, and the mounting plate. The bumper beams assembled at the front and rear of the car use the energy-absorbing box to collapse and absorb part of the impact energy and then transmit the remainder to the longitudinal beam and the passenger compartment through both of the beams to reduce the damage to the car structure caused by the collision. In recent years, high-strength steel has been commonly used in Automotive Manufacturing to process automobile crash beams. Although it can provide good impact resistance and durability, it has a higher weight still, which brings about growth in automobile fuel consumption and emissions. In the context of advocating an environmentally friendly and green lifestyle, the general use of traditional steel deviates from the development trend of lightweight automobiles.

**Figure 1:**
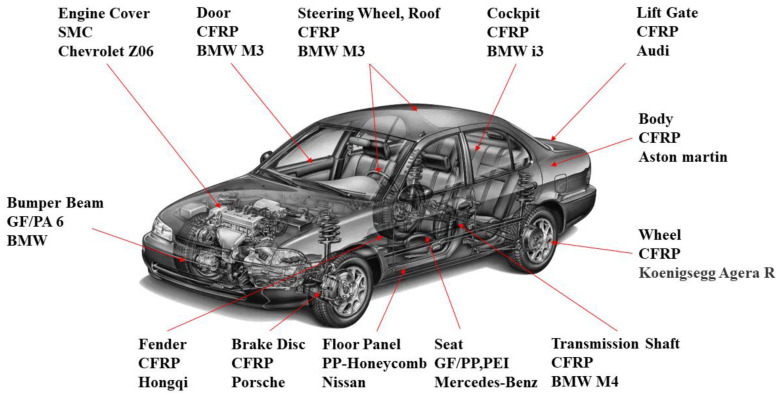
Application of composites in vehicle.

The composite material industry has developed rapidly in recent years, so the forthputting of composite materials with excellent performance to replace traditional materials has become the significant direction for exploring the development of lightweight vehicles, as shown in Figure 1. Xue et al. [1] conducted the research based on a long fiber-reinforced thermoplastic composites (LFT) subject, a low-speed collision finite element model built to design and optimize the structure of the LFT-PP bumper beam. The research demonstrates that compared with the traditional aluminum bumper beam, the new composite bumper beam is not only 17.4% lighter and the manufacturing cost is reduced by 69%, but the energy absorption effect has significantly improved. Jiang et al. [2] obtained the parameters of the CFRP material through mechanical performance tests. They took the mass, specific energy absorption, maximum intrusion, and peak collision force of the bumper beam as objectives, and then optimized the layup of the anti-collision beam by the entropy-based TOPSIS approach. The anti-collision beam is reduced by 76.82%. According to the research of Du et al. [3], through the improvement in the bumper beam-forming process, the weight of the carbon fiber composite beam is reduced by 45%, compared with that of the steel beam, and the ultimate load it bears is reduced to 14.9 kN. According to Cheon et al. [4], through the composite bumper beam to carry out the collision buffer test and performing the static bending experiment, it can conclude that the weight reduction effect of the composites type calls for 30% under the same bending strength. According to Evans [5], the performance parameters were obtained by observing the impact tests of composite bumpers made using different molding methods under a 16 km/h swing obstacle between the scope of −30~60 °C. According to Dacoodi et al. [6], after simulating the low-velocity impact tests of the natural fiber composite bumper beam in Abaqus, the evaluation matrix is formed by analyzing the impact of six characteristics, the deflection, strain energy, weight, cost, manufacture, and the feasibility of ribs, and then determining the double-hat profile (DHP) of the material model that can be used for bumper beams of undersized autos.

In this paper, the use status and development of traditional steel bumper beams are introduced, and then the composite materials suitable for automobile bumper beams are analyzed from two aspects, the thermosetting and thermoplastic. Next, the molding process of the above materials is analyzed. Finally, the performance of the composite bumper beam is verified experimentally by the combination of static/dynamic experiments and a finite element simulation, and then the results are summarized and optimized.

## 2. Material of the Bumper Beam

Steel and aluminum materials are mostly used in the manufacture of bumper beams so that there is a lot of room for the composite materials to expand. The steel bumper beam has a low usage cost, but the collapse distance is short. The bumper beam made of aluminum has a lower density than steel, the weight is lower than steel at the same thickness, and the collapse distance is longer, which has a better energy absorption performance. Composite materials have a higher specific stiffness and specific strength, so composite bumper beams have significant advantages in terms of weight reduction. Some automobile manufacturers utilize glass-mat-reinforced thermoplastics (GMT) to fabricate the rear bumper beam. The comparison of the materials is shown in Table 1.

### 2.1. Traditional Material

**Figure 2:**
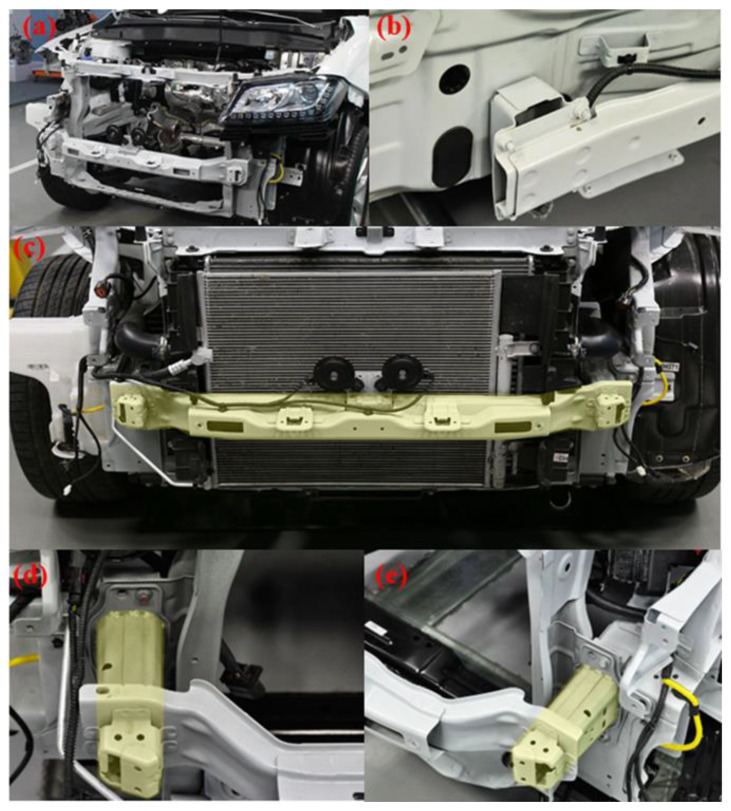
Steel bumper beam and crash box of Haval H2. (**a**) Front frame, (**b**) rear bumper beam, (**c**) front bumper beam and steel buffer plate, (**d**) left crash box, (**e**) right crash box.

There are two main types of materials used in automobile bumper beams. One is materials that achieve lightweight effects by reducing the thickness of the anti-collision beam and improving the mechanical properties of materials, such as high-strength steel material, as shown in Figure 2. Another is material that optimizes the structure to attain a weight reduction by reducing its density, such as carbon fiber composite materials, etc.

AHSS can be further divided into several subcategories based on their properties and intended applications, including Dual-Phase (DP), Transformation-Induced Plasticity (TRIP), martensitic, and Complex-Phase (CP) steels. DP steel, TRIP steel, and martensitic steel (M) are employed in the production of automobile bumper beams. High-strength steels such as TRIP steels [7], processed using manufacturing methods including cold rolling, annealing, or a thermomechanical process, can decrease the thickness of the sheets while increasing the strength. Therefore, the weight reduction in the product is improved. The medium-Mn automotive sheet steels are used in the manufacture of automotive components. Grajcar et al. [8] studied medium-manganese steels with different Mn contents (3 and 5%) and conducted thermomechanical rolling tests of 3.3 mm sheets. The results showed that the steels containing 3% Mn are characterized by good strength and ductility; the increase in Mn content to 5% leads to ultra-high-strength levels. Due to the low production cost of steel materials and the various ways to obtain raw materials, steel is still used as the material for the front bumper beam, as shown in Table 2. Traditional steel bumper beams are generally associated with the body by welding or riveting. Sheets with a thickness varying from 1.5 to 2 mm are often selected to improve the impact resistance of the bumper beam. Through the result of the comparison between high-strength steel and conventional steel, the former has a higher yield stress and tensile strength, as shown in Table 3 [9].

The application of advanced high-strength steel (AHSS) is an important direction for a light weight. The rendition of bumper beams that use high-strength steel is more satisfactory. On the one hand, due to its low hardening index, thickness anisotropy coefficient, and elongation, its formability is insufficient, and on account of the characteristics of the material, the beam is susceptible to corrosion, resulting in a trimmed service life. On the other hand, the thickness of the bumper beam is still heightened, resulting in a massive weight. With the demand for lightweight, some scholars have developed ultra-high-strength steel with a higher yield strength, which has an influential effect on the lightweight, yet it was not put into large-scale use in automobile bumper beams. According to the research of Zeng et al. [10], in simulating a bumper in a high-speed frontal collision, by utilizing composite materials instead of steel for optimization, the weight and HIC of the bumper beam were reduced while ensuring structural safety. Through his experiments, we learn that compared with steel bumpers, composite bumpers have a higher energy absorption efficiency and lighter material weight in a collision. The weight of the beam structure before the optimization is 4.813 kg, and the mass after that is 4.58 kg. The weight of the bumper beam through the lightweight design is reduced by 4.84%, while the peak of the impact energy absorption is 1.36 times that of the steel part.

Aluminum foam and magnesium alloys are also used to fabricate bumper beams as advanced materials. One of the key properties of aluminum foam is its ability to absorb energy; it has the characteristic of obtaining higher strains at lower stress levels [11]. The inimitable property of closed-cell aluminum foam (CCAF) is its lightweight structure which promotes its application in the automotive industry [12]. The application of magnesium alloys in the bumper beam helped to reduce the weight, which contributed to improved fuel efficiency. It was demonstrated that the application of magnesium alloys in bumper beams, crush tips, and intrusion beams can improve the lightweight significantly [13].

### 2.2. Composite Material

**Figure 3:**
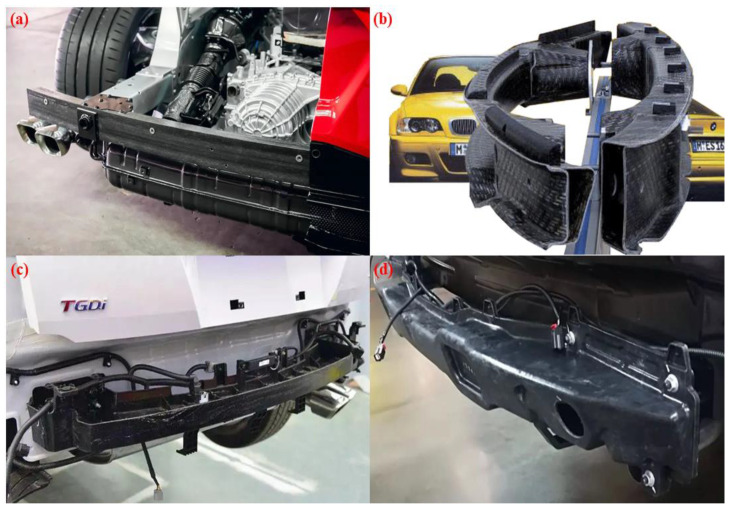
Application of composites in bumper beam. (**a**) Corvette C8, (**b**) BMW M3, (**c**) Hyundai TUCSON, (**d**) GEELY Emgrand X7 Sport.

A composite material is a multiphase solid material composed of two or more substances with different physical or chemical properties. Commodities made from it have the advantages of high quality, high specific strength, high specific rigidity, and high corrosion resistance. However, the composite material also has some limitations and disadvantages, such as environmental sensitivity, a relatively low damage tolerance, a time-consuming manufacturing process, an expensive cost, and limited strength in the direction perpendicular to the fiber orientation. Despite the challenges, composite materials continue to be widely used in the lightweight field of automobiles, as shown in Figure 3.

Thermosetting composites are employed diffusely in a lightweight vehicle due to the outstanding heat resistance and structural stability under compression. According to the research of Zheng et al. [14], through manipulating XB3585 epoxy and T300 carbon fiber piles as reinforcements, the vehicle bumper beam obtained by integral processing is an addition to the quasi-static mechanical property experimentation and through a fatigue testing device. The results are identical to those in the Abaqus simulation. Chen et al. [15] designed the bumper beam structure utilizing T300/5205 carbon fiber composite materials, then established a finite element model and compared it with the steel bumper beam collision test. They studied the consequence of a cross-section and ply sequence on the impact of carbon fiber composite bumper beams and carried out multi-objective optimization on the thickness of the ply using the NSGA-II genetic algorithm. The results showed that the light weight of carbon fiber composite materials reached 64.5%, and the bumper beam still fulfills the stiffness requirements. According to the research of Neelima et al. [16], they compared the crashworthiness performance of steel material and S2 glass fiber-reinforced epoxy resin composites in low-speed collisions through a demonstration. The data acquired through the test are present in Table 4, including parameters such as the material and thickness. The stress of this bumper declined to 727.15 N/mm^2^, and the overall morph reduced to 33.25 mm. The stress reduction rate of the new model is 67.14%, and the total morph reduction rate is 84.25% under the same impact conditions.

For lightweight automobiles, Dixit et al. [17] examined the deformation characteristics and crushing performance of a carbon fiber-reinforced polymer and front bumper components made of steel in a quarter-point impact test. In experiments, they employed simplified components for comparison, making known that the typical collapse mode of a composite bumper is a crash-pot failure and bumper failure due to high stresses caused by the bending deformation after impact. Instead, their plastic deformation is mostly the failure of steel FBCCs components in the quarter-impact test. CFRP is a more effective and lighter material in energy absorption after we dissect the conclusions. Agunsoye et al. [18] enhanced the epoxy composites structure and mechanical properties by carbonized coconut shell nanoparticles (CSnp). They incorporated LY556 epoxy resin with CSnp at a ratio of 5~25% and checked by scanning electron microscopy and a thermogravimetric analysis that counting CSnp at 25 wt% produced the finest value. By analyzing the comparison result of the two Toyota models, it can be obtained that the optimal impact value of the newly developed epoxy resin composite material is 10.5% higher than that of the Big Daddy Model and 37.45% higher than that of the Carina model under the same test environments. Ramakrishna et al. [19] analyzed the properties of natural hybrid particulate fiber composites. The natural fiber of this composite material has a light weight, and its mechanical properties can adjust according to customized requirements, which makes it have competitiveness in the automobile manufacturing industry.

Thermoplastic composites have a low density, high strength, and better designability than thermoset composites. The waste can be recycled, which can decline the material loss and reduce costs because thermoplastic composites can be recycled.

**Figure 4:**
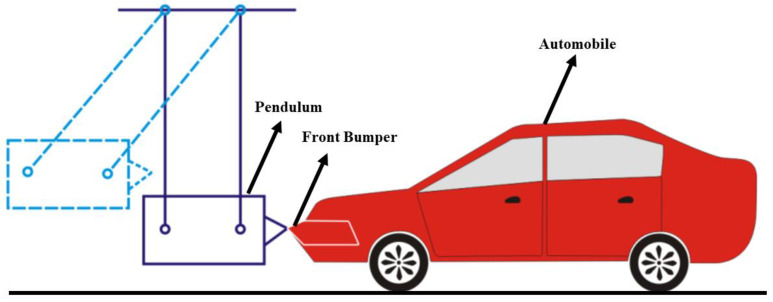
Low-speed impact test [20].

Roopesh et al. [20] designed a low-speed impact examination to compare the crashworthiness performance of bumper beams of SMC, GMT, and aluminum alloy, as shown in Figure 4. The experimental results demonstrate that the performance of the bumper beam of the GMT and SMC are both better than that of the aluminum alloy, and the weight has significantly declined. In addition, the SMC has the characteristics of low cost and convenient manufacture, compared with the GM. Therefore, the SMC is a more satisfactory automotive lightweight material. Grauers [21] employed polycaprolactam, glass fiber reinforced, and the material that is steel reinforced to study the collapse cause of bumper beams. They simulated and trialed the failure mode of bumper beams through a finite element analysis and the amalgamation of dynamic experiments and static tests. Zhang et al. [22] conducted frontal collision experiments on ultra-high-strength steel front bumper beams of different strengths and thickness. They studied the influence of the materials and thickness on the performance of front bumper beams. The experimental results show that the bumper beam with the same strength increases with the expansion of the thickness. In addition, the experiment proves that for the bumper beam with the same cross-section, by employing higher strength materials, it is possible to achieve a good weight reduction effect and obtain a good performance. Belingardi et al. [23] investigated the reusable lightweight material for the bumper beam by exploring the practical application of the GMT, GMTex, and GMT-U in the front vehicle bumper beam. Through a detailed test material characterization, a numerical analysis, and improvements to the GMT using textile fiber and unidirectional fibrous material, they enhanced the tensile and impact properties of the raw material. The traditional beams can be generated with ameliorated materials.

Adesina et al. [24] considered natural fiber and its hybrid fibers during the synthesis of a macromolecular compound matrix and the application of polymer matrices and compared them with traditional glass-mat-reinforced thermoplastic and long fiber-reinforced thermoplastics, and then took hybrid natural fiber to contrast the collision performance. Their experiments concluded that we need to survey nanofiber composites, which cannot bring off the same performance as the GMT or LFRT through the manipulation of a toughener for strength reinforcement.

### 2.3. The High Strain Rate Properties of Materials

The high strain rate properties of a material referred to the strain rate that the material can resist when subjected to a high-velocity impact. High strain rate properties are critical for many applications, such as automotive safety systems and bumper beams.

**Figure 5:**
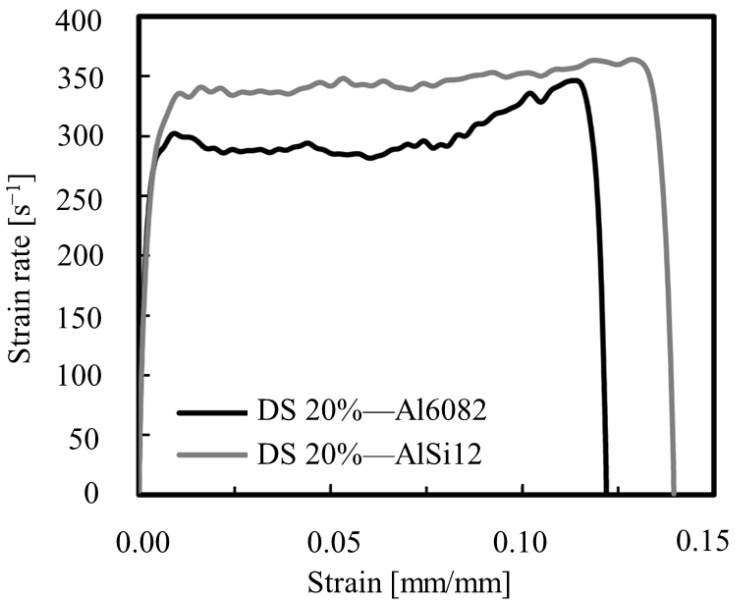
Strain rate–strain curves [25].

In the collision of automobile bumper beams, the high strain rate characteristics of materials play an essential part. The high strain rate deformation process can be considered as the energy absorption of materials to effectively protect the car structure from a collision. When a vehicle crashes, the crash beam is subjected to enormous impact forces, which cause the high strain rate deformation of the material. The bumper beam can absorb a large force and reduce the impact if the material has a high strain rate capability. For traditional materials, the high strain rate properties can be improved by transfer and texture. In addition, we can use composite materials or porous materials. Amaro et al. [25] employed a lost wax casting process to prepare samples having nominal compositions of AlSi12, Al6082-T4, and aluminum foam-filled tubes. They used a split Hopkinson pressure bar (SHPB) to conduct an impact test at different velocities, and the results show that Al6082-T4 foams have a better performance than AlSi12 samples. The Al6082-T4 foams arrangements of the dual-size cell (DS) showed a higher capacity to absorb energy, and the strain rate–strain curves for the Al6082-T4 and AlSi12 foams for the DS 20% are shown in Figure 5.

## 3. Fabrication of Bumper Beam

### 3.1. Fabrication of Traditional Materials Bumper Beam

The traditional fabrication of the bumper beam generally exercises cold pressing that involves extrusion, sawing, bending, milling, and cutting, accompanied by heat treatment, connected by welding or bolting at last. Products will receive surface anodizing, surface spraying, and other processes according to appearance requirements to attain levelness or wear resistance. The cold pressing and casting molding is purified by abrasives, while the components have excellent rigidity. Thus, it is convenient to yield complex figures by a batch that is arduous to manufacture by general crafts. The mature production line enables a towering fabrication efficiency and maximizes the utilization of materials, ensuring the consistency and stability of the process. However, its defects are noticeable. Due to the independent procedure, the stamping crafts have a low integration. In addition, the process is complicated, which means the process can not satisfy the standards when the units have extremely high precision requirements.

**Figure 6:**
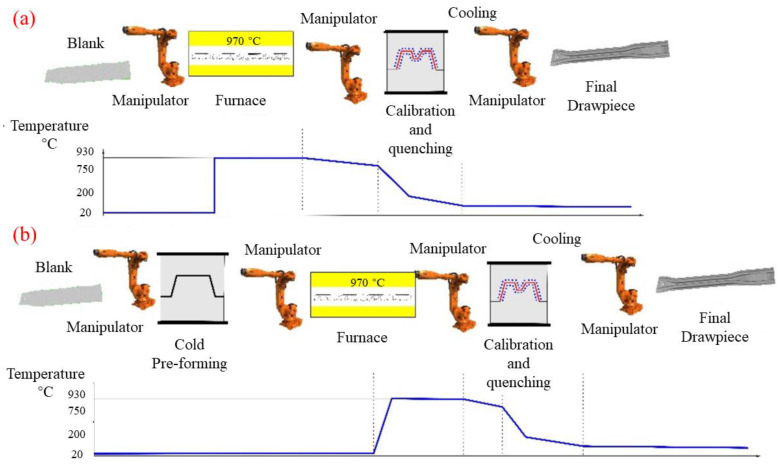
Scheme of the hot stamping process: (**a**) direct hot stamping and (**b**) indirect hot stamping [26].

Hot stamping has the advantages of being fully automated, having good formability, and having relatively high precision. The recent challenges faced by this technology are the process optimization, cost and material limitation, application for large-sized car body parts, etc. Hot stamping can produce high-strength steel automotive structures and the process consists of two methods: direct and indirect, as shown in the Figure 6 [26].

### 3.2. Fabrication of Composite Materials Bumper Beam

**Figure 7:**
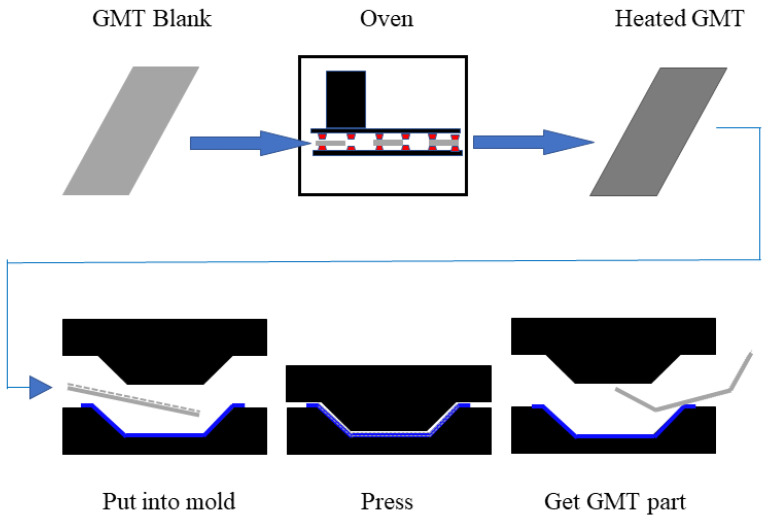
Molding process of GMT molded part [27].

Fabrication and product performance are intently allied. For example, composite materials and processes demand they be in accordance and support each other. The fully developed fabrication method guards the excellent performance of composites. In the 1960s, the United States began developing glass-mat-reinforced thermoplastics (GMT), and it was employed in mass production in the 1990s [27]. The GMT molding process is shown in Figure 7. GMT is used in the fabrication of automobile rear bumper beams in vehicle parts production. The molding cycle of GMT is 30–40 s. GMT bumpers have been in continuous service in various passenger cars for more than three decades due to the utilization of regenerated, short molding cycles and high production efficiency. The front bumper of the 1984 Chevrolet Corvette was the first to use GMT composites. After the Corvette bumper, the GMT design launched on Cadillac DeVille, Brougham sedans, etc. The GMT was used for the first time on the rear bumper of the Ford Continental DN9 sedan. Over 16% of passenger cars were equipped with GMT bumper beams globally in the 1990s. The service of GMT presents opportunities for a light weight and cost reduction.

**Figure 8:**
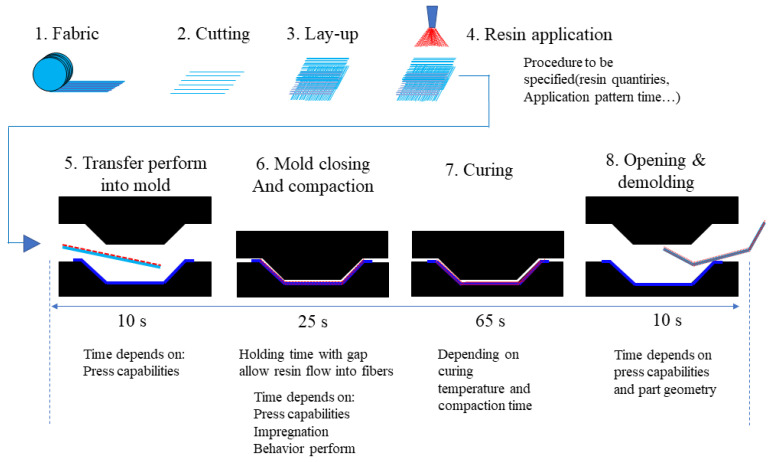
Wet molding process (MOMENTIVE, USA) [27].

Wet compression molding (WCM, wet pressing, liquid compression molding) is a novel measure of composites that produce molding, and its molding cycle is around 110 s. KraussMaffei was the first to implement it, then the Cannon from Italy and Dieffenbacher from Germany carried out the development of wet molding, respectively. The process of wet molding is shown in Figure 8 [27]. It is characterized by employing dry fibers and liquid resins as raw materials instead of fiber-reinforced resin prepreg. The primary steps of wet compression molding to manufacture the bumper beam is first to design the carbon fiber cloth in the light of the figure, then infiltrate the fiber cloth into the abrasives, and clamp, solidify, and apply pressure to it, thus gaining a semifinished product. After opening the pore and edge trim, we obtained the bumper. The WCM fabrication has advantages that produce swiftness, efficiency, and automation. It was employed in parts of BMW and Audi vehicles widely for a series.

**Figure 9:**
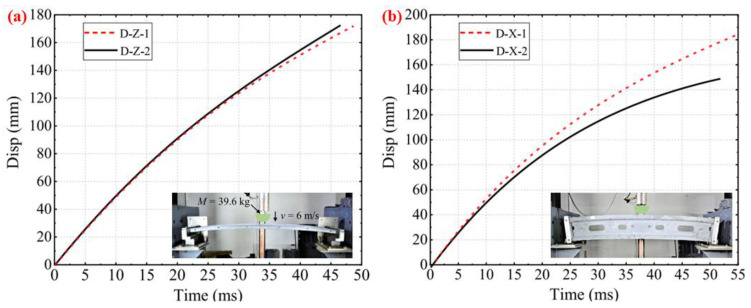
Displacement curves: (**a**) Z-directional tests, (**b**) X-directional tests.

Ren et al. [28] took WCM to utilize a carbon fiber composite bumper and confirmed the performance by conducting a frontal crash test (40%). The conclusions of the comparison test with the function of the steel bumper beam show the peak intrusion amount is 16.8% below that made of steel, yet the energy absorption is 57% superior. Liu et al. [29] used a lightweight CFRP header rail fabricated by WCM to conduct drop-weight impact tests. Then, they compared the failure modes, force responses, energy absorption characteristics, and structural responses to different directional loads. The experiment results demonstrated that the impact performance of the specimens under a Z-directional impact was better than under an X-directional impact with the same loading magnitude, as shown in Figure 9.

**Figure 10:**
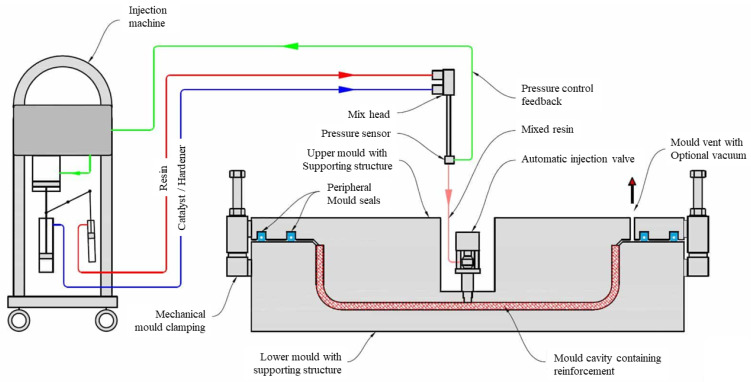
RTM molding process principle (composite-integration.co.uk/resin-transfer-molding).

**Figure 11:**
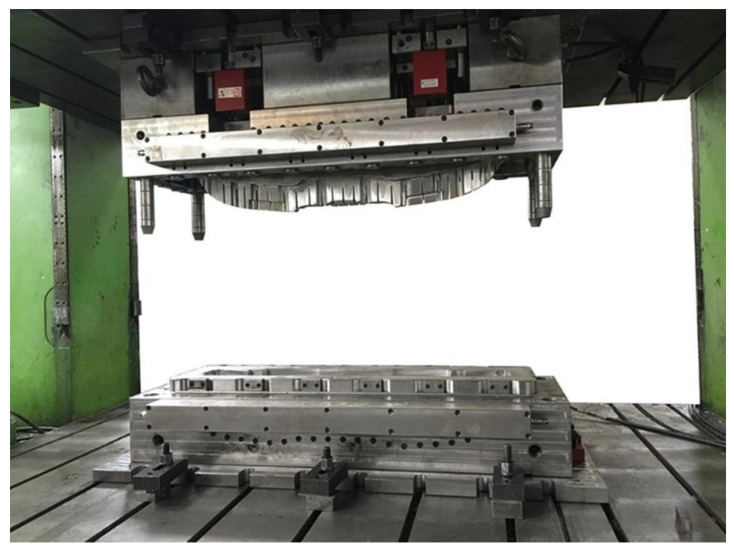
Model of bumper beam with RTM.

In addition to WCM, composite bumper beam manufacturing can employ resin transfer molding (RTM), which is characterized by the divides of the procedures, impregnated resin, and the curing molding process, design, and manufacturing of reinforced fiber, and its molding cycle is 20–80 s. It has a precise thickness accuracy, a smooth exterior, and a low production cost. The process of manufacturing the bumper beam is foremost to arrange the fiber cloth, and then the fiber is spread into the abrasive and soaked, accomplished after the resin curing, as shown in Figure 10. The RTM crafts forcefully increase the liquidity and wetness of the resin fiber and significantly heighten the fiber contents, which is the origin of the improvement in the properties. In the past years, RTM fabrications were applied increasingly in the production of automobiles, as shown in Figure 11. The development of the RTM has attracted increased attention. Zhao [30] adopted the RTM crafts and developed a fast-curing resin system in accordance with it, which warrants that it can fill the numerous demands of composite components. They took 1B2MZ, 2E4MI, and API to be compounded, respectively, with IPDA as curing agents, thereby determining the technical parameters of the three fastest curing formulations. Finally, GFRP composites were prepared by VARTM and captured the formula with an excellent performance through experiments. Wang et al. [31] simulated the resin flow model of the composite through PAM-RTM software and others, and the results certificated that owing to the strict precision demands of the RTM process, we should cut down the number of closed-angle structures. The chosen material should have adequate intensity, and the constitution of the mold should be designed logically. Park et al. [32] manufactured flax/vinyl ester-applied composite material with RTM for analyzing the structural design of the automobile bonnet with a natural flax composite. The thickness of the flax/vinyl ester panel was 6 mm and the stacking sequence was [±45]_6_, and the results of the impact test show that the bonnet structure designed with RTM is acceptable.

Additive manufacturing (AM), known as 3D printing technology, has the potential to promote the development of the automobile industry by producing lightweight, complex, and customized parts and structures. AM helps reduce the car weight and improve the fuel efficiency and performance. The additive manufacturing of polymer and metal tools is reducing the lead times for prototype development [33], quickly and inexpensively creating concept models of automotive components and allowing for faster design iterations and tests. In addition, it can repair damaged parts by producing custom parts, which facilitates the repair and replacement of damaged components. Additive manufacturing does not need molds and metals when making supplemental parts such as rubber, plastic, and a cylinder head, which can effectively simplify complex mold processing, significantly save human and equipment resources in the manufacturing process, and reduce the cost investment [34].

## 4. Performance of Lightweight Bumper Beam

### 4.1. Industry Regulations and Performance Test Methods

**Figure 12:**
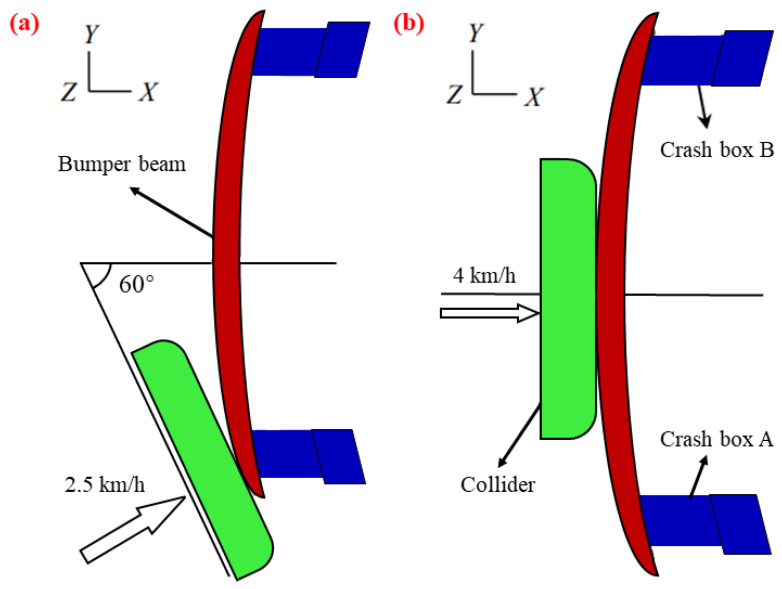
Tests specified of (**a**) angular collision and (**b**) center collision in GB17354-1998 [37].

Vehicle collisions are divided into frontal impact tests, side impact tests, and rear-end collisions, among which frontal collisions account for 4.9% of traffic accidents, the highest proportion. According to US insurance companies, low-speed collisions account for the immense majority of accidents. A low-speed collision is defined as a collision that occurs at speeds under 15 km/h [35]. The standard index of domestic automobile bumpers is GB17354-1998, which stipulates the details that the remaining parts and systems of the automobile, save the bumpers, will not be destroyed in a low-speed collision. The specifics of GB17354-1998 are shown in Table 5 and Figure 12.

Currently, three kinds of automobile crash standards are widely adopted, as shown in Table 6. So far, the analysis methods of the bumper performance comprise experiments, a finite element simulation, and the mass-spring-damper system. With the improvement in the finite element theory system in recent years, the finite element analysis method can obtain more accurate data simultaneously using the least cost.

**Figure 13:**
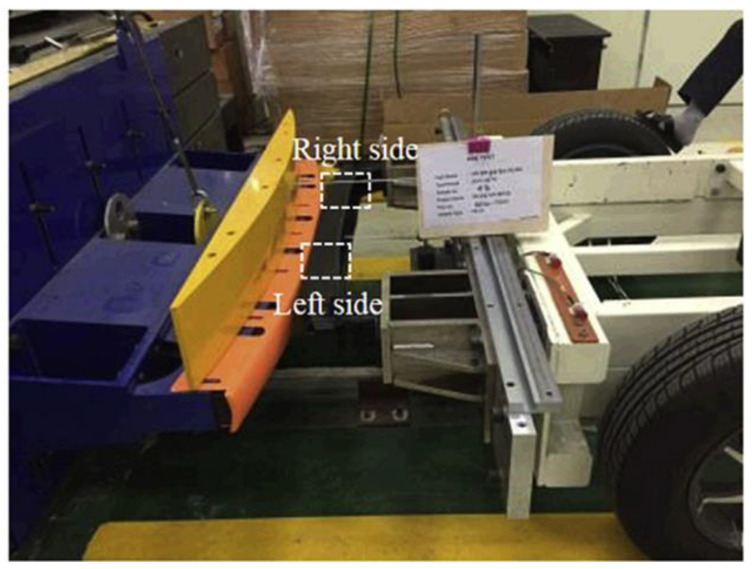
The glass fiber reinforced thermoplastic polypropylene (GFPP) bumper beam impact test [36].

**Figure 14:**
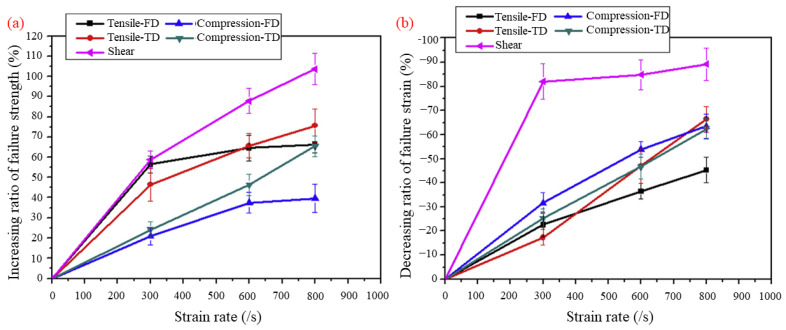
Experimental results for strain rate-dependent mechanical properties of GFPP, including the fiber direction, transverse direction, and shear: (**a**) increasing ratio of ultimate failure strength; (**b**) decreasing ratio of failure strain [36].

Kim et al. [36] studied the effect of the high strain rate on dynamic tensile, compressive, and bias-extension shear behaviors using the split Hopkinson pressure bar (SHPB) equipment. They analyzed the strain rate failure mode of each specimen fracture. In addition, bumper beam impact simulations were performed using LS-DYNA and compared with those of quasi-static properties. Figure 13 shows the condition of the impact test. Figure 14 shows that the effects of the strain rate were most evident in the results of the bias-extension test, where the highest increase in the shear strength (103%) and the initial shear modulus (89%) were observed at 800 s^−1^ of the strain rate. The results show that the increasing ratio of the failure strength in the matrix-dominant properties are increasing continuously to the strain rate, while that of the fiber-dominant properties seems to be saturated from the 600 s^−1^ of the strain rate.

### 4.2. Theory of Crash Simulation Analysis

The finite element analysis is a calculation method that generally applies in engineering analysis. It divides the object from a whole into innumerable tightly linked individuals by organically associating software, mechanics, and mathematics. The calculations are conducted during a simulation, and then there is an integration of data; thus, we can gain characteristics of the whole objects. The finite element analysis method is flexible and easily handles structures with complex figures and multiplex properties; prompt, it has diverse applications and it can smoothly manage inhomogeneous materials, anisotropic materials, and nonlinear stress, strain, and compound boundary conditions in a stress analysis conundrum [37].

The vehicle crash tests contain presets for a large displacement and immense strain. During the collision movement, it is formidable to portray the process accurately owing to the mutual extrusion or clash of the parts. Theories describing high-speed collision phenomena include the Euler method, the ALE (Arbitrary Lagrangian–Eulerian) method, and the Lagrange method. The Euler method is widely used in fluid mechanics issues. The ALE method is for dealing with fluid–solid interactions and is suitable for describing high-speed collision phenomena, but it is hard to implement in programming and engineering applications. The Lagrange method is the most mature method for describing the collision behavior of solids [38]. The finite element method described by the Lagrange method can deal with complicated boundary conditions and complex material constitutive relations during high-speed collisions. Therefore, the nonlinear finite element method usually adopts the Lagrangian theory, which is the most employed one in automobile crash simulation analyses.

### 4.3. Performance Test Experiment of Composites Bumper Beam

Liu [39], respectively, holds low-speed collision tests and three-point bending experiments on their CFRP bumper beams. They employed the optimized CFRP beam for crash tests and installed the beam and energy-absorbing box at the front side of the car, which means it was settled on the facade unit. The box is made of aluminum, and the section shape of the beam looks identical to the shape “B” and is reinforced by riveting. Before the experiment, a scrutiny point on the box was marked. After, for the box itself, the deformation at the point is rather tiny, so the influence of the energy-absorbing box on the performance of the beam can be ignored. The peak of the impulse force is 24.908 kN, and the maximum deformation is 54.4067 mm at 83 ms. Park [40] established the dynamic equivalent method to simplify a nonlinear dynamic crash model and proposed a simple bumper crash analysis model as well as a new IIHS bumper model in their trials. By utilizing the finite element analysis and contrasting the results, they found that the maximum displacement deviation of the two models was less than 1.95% during the first kind and less than 13.2% during the impact of the new IIHS. Park [41] employed a dynamic equivalent beam optimization method instead of the traditional method. They constructed dynamic models for different clash conditions and used the nonlinear finite element analysis to check the accuracy. They adopted the bumper’s rib into the calculation and developed a dynamic impact beam model. This approach supports reduced time costs and trial costs.

**Figure 15:**
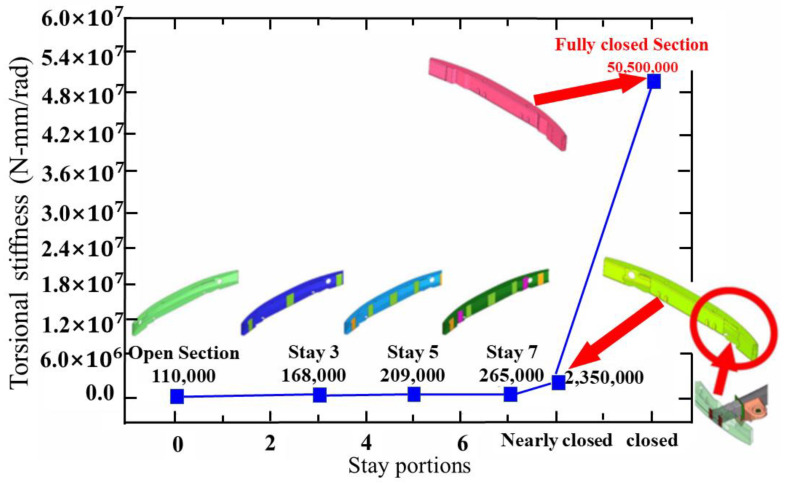
Effect of stay portions on torsional stiffness [42].

Kim et al. [42] analyzed the effect of the torsional stiffness of the impact beam of the bumper, taking it as a gambit to lower the weight of beam. They concluded that increasing the section height was the most effective means for controlling the section height, beam thickness, crash brake, and torsional stiffness. The simulation results in Figure 15 show how the torsional stiffness of a bumper impact beam is affected by the structure; in an impact beam with a fully closed section, the torsional stiffness was about 500 times that of an open section. In addition, they compared the capabilities of traditional steel bumper beams with high-strength steel beams for investigating the potential expansion capacity. Muhammad Nasiruddin S et al. [43] explored the factors touching the energy absorption of bumper beams. A lot of discussions conducted showed that natural fiber polymer matrix composites were applied in vehicle parts, yet the research on the bumper beam structure is still advancing. It means that the applicability of such composites in bumper beam structures desires further investigation. Lorenzo et al. [44] discussed the design of a metal matrix composites bumper and analyzed the feasibility of applying laminated steel/composites beams as an alternative to conventional materials in the front bumper system, supplanting them with composites in the 89 Cadillac C sedan. Compression-formed continuous glass-reinforced polypropylene was evaluated by the application of the CAE. The total weight of the laminated steel/composite system was 88.96 N. The employment of the laminated steel contributed to a more than 50% reduction in the total weight.

### 4.4. Finite Element Analysis of Bumper Beam

In a finite element analysis, the size and number of meshes may influence the accuracy and convergence of the simulation results. If the mesh size is too large, the finite element model will reduce the agreement with the actual conditions, resulting in errors. On the other hand, if the mesh size is too small, the computation time will increase significantly, and the results will be greatly affected by numerical errors. For this reason, choosing an appropriate mesh size for the specific problem is especially important to obtain reliable results.

Numerical simulations are widely used in vehicle design and crash assessment, while material models affect the elasticity and damage response under both quasi-static and high-velocity loads [45]. The constitutive model can describe the relationship between strain and stress, while the failure criterion can predict the conditions in which the material will fail. Both are essential for vehicle design and analysis. Duan et al. [46] studied the effect of the strain rate of auto parts using LGFRP materials. They analyzed the crashworthiness and energy absorption effects by the visco-plastic constitutive model. The model agrees with the experimental results well. Yang et al. [47] employed the nonlinear explicit FE code LS-DYNA for simulations and the bumper beams (HSS DP600 and aluminum alloy AA6064-T4) were modeled using Mat 24 (piecewise linear plasticity). Çam et al. [48] carried out the numerical studies by utilizing the commercial FE software LS-DYNA. They selected a piecewise linear plasticity material model for a steel bumper beam and crash box. They employed an enhanced composite damage model for the IM7/8552 fiber prepreg/epoxy composite bumper beam and used the Chang criterion for the failure.

The Johnson–Cook model [49,50] and Zerilli–Armstrong model [51] can be selected for steel and aluminum material under high strain rates. At low strain rates, the material behavior is typically characterized by linear elastic behavior. The von Mises yield criterion and Tresca criterion [52] can be used to describe the behavior of steel material. For composites, the commonly used failure criterion types include the maximum stress criterion [53], maximum strain criterion, Tsai–Hill criterion, Tsai–Wu criterion, Hashin criterion [54], and Puck criterion [55].

Xiao et al. [56] conducted a finite element analysis test on a steel bumper beam under a low-speed collision. They meshed the structure with HyperMesh and set the whole auto as a collider; the mesh size of the beam was 10 and 15 mm for the panel. Assigning the material property with steel, the density was 7.85 × 10^−9^ t/mm^3^, Young’s modulus was 2.1 × 10^5^ MPa, and Poisson’s ratio was 0.3 in the MAT24 material model. By giving the collider an initial speed of 1111.11 mm/s along the positive direction of the X-directional, a low-speed front collision was thus conducted. After the calculation, the energy altered the curve drawn in HyperGraph. Because the curve was smooth and the hourglass energy maximum was 11.033 J, which is less than 5% of the total, this makes the results highly reliable. The peak impact force of the energy-absorbing box section was 15.827 kN, and the minimum intrusion amount was −67.6731 mm.

Li [57] improved the function of the composites beam through structural optimization. They utilized a finite element model under a low-speed crash for testing. The section thickness of the composite bumper was 2.4 mm. To maintain an efficient calculation, they meshed the thin shell structure. It involved 25 627 elements and 26 943 nodes. The mesh was 5 mm, and the mat55_54 material constitutive model was recorded in the LS-DYNA database as the property of the carbon fiber composites. Intending to simulate the crash condition, they took the pendulum acts as a collider. The head of the collider was made of high-tension steel. The pendulum hit the bumper beam at 4 km/h. The end of the energy absorption box constrains the degrees of freedom in the 1, 3, 4, 5, and 6 directions. The interaction of the reference surface between the box and the pendulum was of the surface-to-surface type, and the others were an automatic single surface. In the experiment, three kinds of composite bumpers with different sections were compared, and the conclusion is that the section shape “n” had the best performance by adding on the square section. Hambali et al. [58] analyzed six different composite materials by the AHP method, employing 8 major factors and 12 sub-factors to determine the most suitable material for application in bumper beams; the study demonstrated that glass-reinforced fiber epoxy resin is the most appropriate.

K. Praveen Jerish et al. [59] created a composite bumper system by confusing Oobleck’s non-Newtonian fluid and HDPE materials. The test utilized its thickening properties to curing fluids in HDPE pipes. The deformation history by the developed non-Newtonian fluid bumper was percipient compared to the current bumper. It had less structural harm and fewer wrinkled areas. Karthikeyan et al. [60] studied the performance of different bumper materials and proposed the development of the bumper for the i10 vehicle. Such as the ABE, E glass fiber epoxy resin, and polyamide-30% glass fibres, they utilized ANSYS Explicit for the simulation analysis and considered two cases: a full-frontal impact and a half-impact on the baulk. They evaluated the crashworthiness of the upgrade beams by analyzing and optimizing the simulation.

**Figure 16:**
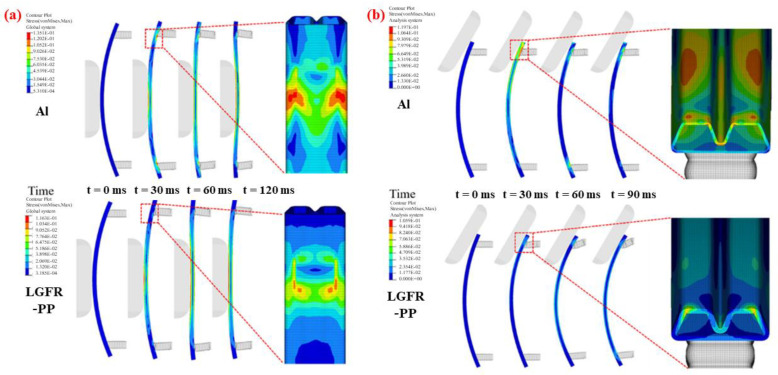
Stress distribution of the LGFR-PP and Al bumper beams under the (**a**) longitudinal and (**b**) angular impact conditions [62].

Wang et al. [61] used the CNPR structure as the suspension bumper of the vehicle and discussed the load–displacement curve of the NPR bumper. In addition, the influence of the structure and materials on the load–displacement curve were specifically investigated. They used two-dimensional quadrilateral shell elements instead of three-dimensional elements because of the small layer thicknesses and demands for calculation efficiency. It concluded that only the number of elements and plies could affect the displacement of the NPR bumper. Xue et al. [62] prepared and tested an LGFR-PP specimen, and then obtained the Poisson’s ratio of the material. Finite element models of aluminum alloy and LGFR-PP bumpers were established to obtain their peak impact force, maximum intrusion, and energy absorption values under longitudinal and angular low-speed impact conditions. They employed the No. 24 material card *MAT_PIECEW-ISE_LINEAR_PLASTICITY to describe the mechanical properties of the LGFR-PP materials. Figure 16 indicates that the Al bumper beam shows more concentrated stress than that of the LGFR-PP, under either longitudinal or angular impact conditions. The results indicate that both of the bumper beams were not damaged in the low-velocity impact under both conditions.

### 4.5. Optimization of Bumper Beam

**Figure 17:**
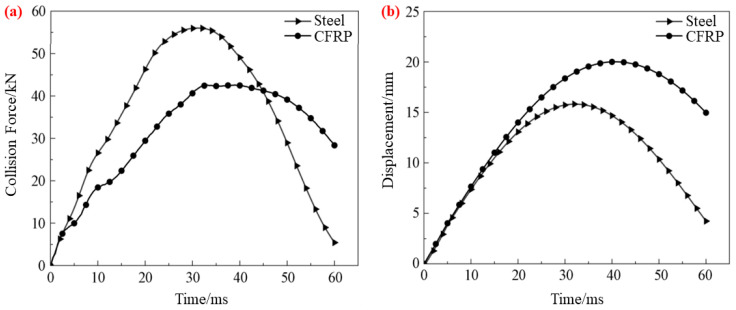
(**a**) Displacement–time curve. (**b**) Collision force–time curve [64].

Huang [63] established a three-point bending finite element model to explore the bending strength of the bumper by studying the low-speed beam accident of a passenger vehicle. They tested the crash beam resistance by a pendulum impact test. They tested the crash beam according to low-speed crash regulations under different crash conditions and optimized the formation. By setting the thickness of point 4 as a variable, the bending strength and low-speed collision performance of the bumper beam lowered the weight. By using the aluminum alloy bumper beam as the object, the intrusion peak value under the ultimate working condition demanded being below 6 mm. To achieve a light weight, the weight of the beam should be less than 25 kg, and the peak of the reaction force should be more than 35 kN. Using the optimal Latin hypercube experiment, 21 groups of data were randomly received, and a response surface was established to solve it with a multi-objective particle swarm optimization algorithm. The conclusion reveals that the optimized bumper beam can function in various conditions while achieving a light weight. Zhang et al. [64] redesigned the magnitude of the bumper beam after considering the structure and cost of the carbon fiber composite beam comprehensively. They established a mathematical equation with the thickness as a variable and the maximum displacement and collision force while taking the energy absorption as an optimization goal, as shown in Figure 17. By utilizing the optimal Latin hypercube design (LHD) to extract 10 samples, the HyperKriging method was employed to form the samples in HyperStudy and then the genetic algorithm (GA) was applied to optimize them; the optimal ply thickness was 0.192 mm, and the total thickness was 3.072 mm.

Hu et al. [65] studied the lightweight and crashworthiness of bumpers, and they used LS-DYNA to simulate carbon fiber-reinforced plastic bumpers instead of high-strength steel. In addition, they studied the energy absorption capacity and dynamic response characteristics of carbon fiber-reinforced bumpers and compared them with steel bumpers. The results indicated that the carbon fiber-reinforced plastic bumper beam had a better energy absorption capacity and dynamic response characteristics. The weight was significantly reduced by nearly 50%. Beyene et al. [66] found that the subsystem of the bumper is the main structure, with an energy absorption function during low-speed impact. The component of this subsystem is the transverse beam, which is usually made of steel, which is contrary to the automotive subsystem’s light weight but can fixed by composites. Serkan et al. [67] used HyperMesh to create a bumper beam model with five shapes of sections but the same weight. Because bumper beams are invalid in different ways under different obstacles, pole and protective impact tests were investigated. The test results show that the figure of the section has a significant effect on the crashworthiness, and the model has a different performance on walls and poles.

**Figure 18:**
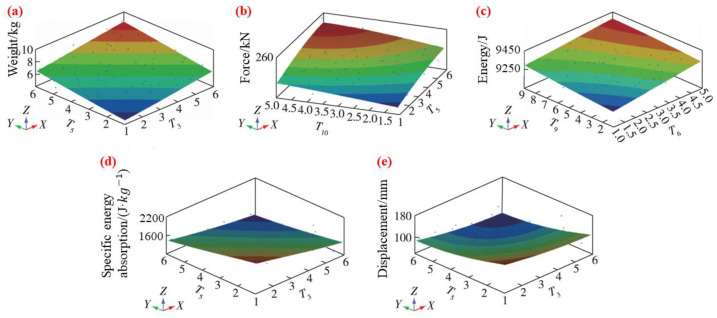
Response surface model of (**a**) weight, (**b**) force, (**c**) energy absorption, (**d**) specific energy absorption, (**e**) displacement of front crash [68].

Zhong et al. [68] studied the optimization of the performance of the bumper in the case of a low-speed collision. They employed the number of plies of the beam and the thickness of the remaining components as variables, and then simplified and established 13 models of different sizes. The response surface was fitted by the Moving Least Squares Method (MLSM) in HyperStudy, the integration points were screened, and the response surface model was obtained after two DOE calculations, as shown in Figure 18. They used the Latin hypercube sampling method to extract 20 sets of data, verified the rationality of the model by calculation, and at last, used the multi-objective genetic algorithm (MOGA) for optimization. They obtained the Pareto diagram by software calculation. From this diagram, the weight of the optimized composite beam was 3.145 kg, which is 13.9% lower than 3.653 kg. The energy absorption ratio was 1854.82 J/kg, which is 15.4% higher than the 1607.72 J/kg of the original bumper beam, and the lightweight effect of the optimization was significantly improved.

Charkha et al. [69] employed sandwich structures to enhance passenger car crash beams. They designed a new composite sandwich structure material with a glass fiber chopped strand mat, core mat, and epoxy resin. This material uses handmade crafts to prepare the composite material; the manufacturing cost is acceptable. By conducting impact experiments, they tested its performance and conducted the CAE verification. The weight of the prepared bumper with the same thickness was reduced by 38.52%, and its cost was reduced by up to 58.33%. The beams ensured that the structure could absorb most of the kinetic energy during the impact. Wang et al. [70] used the building block method to design the impact resistance experiment and reproduced the 3D micromechanical material constitutive relation as a customized subroutine (UMAT) in ABAQUS/Standard. They used a multiplies approach to reliably predict both the mechanicals and deformations and accurately capture the mechanical behavior of the matrix and fiber. Through a validated finite element model (FEM) and a material constitutive model, they investigated the cost and crashworthiness characteristics of an EV composite bumper subsystem at the macroscale. Gil et al. [71] considered that EPP foam is repeatedly applied to absorb energy in bumper systems, but the foam is not sufficient to absorb waves in the interior space. Therefore, the employment of folding bumpers made of engineering plastics can reduce injuries more effectively. They used an indirect method, namely the correlation of the dart drops impact test and the finite element analysis. Finally, an optimized energy absorber made of plastic was proposed, and the measures to strengthen the safety were explored through the study of its model. Chen et al. [72] designed the cross-section of the anti-collision beam, then divided the anti-collision beam into optimized parts, and analyzed the materials and thicknesses of the different parts. They used the radial basis function neural networks approximation model combined with the elitist non-dominated sorting genetic algorithm II to perform the multi-objective optimization on the anti-collision beam. The results show that the multi-material, variable-thickness anti-collision beam can achieve a lightweight rate of 45.45% and has an excellent performance.

## 5. Prospect of Development of Bumper Beam

Nowadays, there are contents worthy of research in the design and optimization of lightweight structures, such as the material selection of bumper beams, size, thickness of bumper beams, shape optimization of cross-sections, etc. It is necessary to consider the combined effect and use the method of combining the experiment and simulation. Tanlak et al. [73] studied the shape optimization of the bumper beam under impact conditions similar to the EuroNCAP test. They obtained diverse optimal shapes by selecting different values of the weight factors in the objective function. Zhang et al. [74] used topology optimization and size optimization methods to improve the structure of the bumper beam, the crash box, and the front section of the front longitudinal beam. The mass of the optimized bumper beam was 1.278 kg, which is 1.351 kg lower than the original structure. By incorporating structural foams, hollow sections, and sandwich structures into the design of bumper beams, it could be possible to reduce the weight of the bumper beam while still maintaining its strength.

With the development of lightweight technology, magnesium will receive more attention. The application of excellent high-strength steel, composite materials, and other advanced materials and advance processing technology can remarkably reduce the weight, thereby achieving a light weight. When designing the bumper beam, in addition to the cost of raw materials, the time cost and labor cost of the fabrication should be considered. The cost is one of the causes why many of the current high-performance materials are not widely used. Discovering an acceptable balance between the usage cost and performance is one of the ways to promote the development of lightweight structures. The life cycle of most automotive materials covers from raw materials to the recycling and disposal [75]. Carbon fiber composite materials and lightweight metals such as aluminum alloys and magnesium alloys have relatively high recycling rates. The recovery methods of the CFRP include incineration, mechanical recovery, chemical recovery, and so on. Alloy materials can be recovered and reused through physical and chemical methods.

## 6. Conclusions

(1) From a lightweight perspective, the best choice is to use high-strength steel and composites with a better performance and lower cost to improve the structure of bumper beams. With the gradual improvement in composites technology in China in recent years, a tendency toward the usage of composite materials to produce vehicles to attain a light weight was formed, which means that the lightweight design of automobile structures is a critical study direction. This paper discusses the benefits of lightweight composite materials compared to traditional bumper beams from the material itself. The experimentations listed prove that the characterization performance of the bumper beams is better than that of the steel and aluminum alloy after using the composites, and the consequence of being lightweight is remarkable. The future development prospects of thermoplastic composites are more profitable than thermoset composites due to their intense designability and being environmentally friendly.
(2) From the perspective of fabrication, the process of traditional bumper beams is complicated and accompanied by the waste of materials. The fabrication of composite bumper beams tends to develop with more research investment in recent years, such as the GMT, RTM, and WCM. This procedure can create products with an excellent performance and can strengthen the capability of the bumper beam by customizing it. Straightforward operation and automation are advantages.
(3) The performance of the composite bumper beam in the application is optimized and experimented with in an experimental analysis and finite element analysis. The composite bumper beam has better impact resistance and a higher class of lightweight in the test of an impact compared with that of steel. The cost of the investment in composite materials study is relatively high in the initial stage, but it will decrease with the improvement and maturity of the process. After that, the application of composite materials in an automobile lightweight will be additionally extensive. The development and popularization of new energy vehicles advance the market for composite materials in lightweight automobiles. With the market enlargement, using composite materials for lightweight vehicle structures will be a concern for manufacturers.

## Figures and Tables

**Table 1:** The comparison of materials.

Materials	Density (g/cm^3^)	Young’s Modulus (GPa)	Poisson’s Ratio (ν)	Yield Stress (MPa)	Flexural Stress (MPa)	Flexural Modulus (GPa)	Tensile Stress (MPa)
Conventional steel	7.8–8.0	200–210	0.27–0.30	250–500	250–500	200–210	200–500
Advanced high-strength steel	7.8–8.0	210–300	0.27–0.30	500–1500	500–1500	200–300	500–1500
Aluminum alloys	2.7–2.9	60–80	0.3–0.4	200–500	200–500	70–80	200–500
Glass fiber-reinforced polypropylene	1.1–1.4	2–3	0.30–0.35	100–300	100–300	2–10	100–300
Carbon fiber-reinforced polypropylene	1.1–1.4	2–4	0.30–0.35	200–400	200–400	2–4	200–400
Glass-mat-reinforced thermoplastics	1.1–1.4	2–3	0.30–0.35	100–300	100–300	2–3	100–300

**Table 2:** Steel front bumper beam thickness of different vehicles.

Brand	Model	Year	Thickness/mm
Volkswagen	Teramont	2021	2.50
Tesla	ModelX 90D	2016	2.20
Volkswagen	Passat	2019	1.84
Cadillac	XT5	2016	1.72
BYD	Qin 100	2017	1.60
GAC MOTOR	M8	2021	1.57
Audi	A3L	2021	1.55
Mercedes-Benzes	GLS	2020	1.53
Ford	EDGE	2021	1.40
Honda	CR-V	2021	1.40
Mercedes-Benzes	Smart Forfour	2016	1.50
Honda	Accord	2018	1.50
Ford	TAURUS	2017	1.44
HAVAL	H6	2016	1.30
TOYOTA	VIOS	2019	1.23
TOYOTA	Future Toyota 86 Concept	2017	1.10

**Table 3:** The parameters of front bumper beam made of high-strength steel (DP1400).

Part	Density (ρ)/ (kg·mm^−3^)	Elastic Modulus E/GPa	Poisson’s Ratio (ν)	Yield Stress/Mpa	Weight/ kg
Front bumper beam	7.86 × 10^−6^	200	0.28	1000	3.989
Energy-absorbing box	7.83 × 10^−6^	200	0.3	414	1.307

**Table 4:** Stress and displacement of varied thickness bumper with S2 glass fiber epoxy material and steel material at 8 kmph (S2 glass fiber epoxy/steel).

Thickness (mm)	Stress (MPa)	Deformation (mm)	Weight (kg)
2/2	2532.4/2278	251.1/104.17	6.8/21.8
3/3	1764.4/1736.3	119.17/51.664	9.4/29.8
4/4	995.2/1012	64.889/28.446	12/37.8
5/5	737.13/742.62	40.503/17.895	14.6/45.8
6/6	549.55/555.83	26.864/11.893	17.2/53.8
7/7	439.29/442.51	18.774/8.3082	19.8/61.8

**Table 5:** Front and rear protective devices for passenger cars (GB17354-1998).

Facilities	Colliders and Moving Walls	
Methods	Center collision	Angular collision
Speed	4 km/h	2.5 km/h
Reference height	445 mm	445 mm
Content brief	All parts are in good condition except the bumpers	

**Table 6:** Bumper beam regulations in various countries.

Contents	Canada CFVSS 215	The US FMVSS 581	The EU ECE R42
Collider center collision	8 km/h	4 km/h	4 km/h
Collider angular collision (60°)	4.8 km/h	2.5 km/h	2.5 km/h
Content brief	Vehicle is usable	Bumper beam damaged only	Bumper beam damaged only

## Data Availability

Not applicable.
